# Ingestion of 14 Hearing Aid Batteries in an Adult Patient

**DOI:** 10.7759/cureus.64354

**Published:** 2024-07-11

**Authors:** Breanna M Jomsky, Hiren J Patel, Travis Smith

**Affiliations:** 1 Medicine, Lake Erie College of Osteopathic Medicine - Bradenton Campus, Bradenton, USA; 2 Medicine, Nova Southeastern University Dr. Kiran C. Patel College of Osteopathic Medicine, Fort Lauderdale, USA; 3 Clinical Curriculum Integration and Assessment, Lake Erie College of Osteopathic Medicine - Bradenton Campus, Bradenton, USA

**Keywords:** colonoscopy, ileum, cecum, foreign body ingestion, hearing aid battery ingestion

## Abstract

Battery ingestion is not a common occurrence in adults. When it occurs in patients of any age, prompt action might be necessary, depending on the type of battery ingested, to prevent damage to the gastric mucosa that is involved in important secreting and absorbing functions required to maintain homeostasis. A 61-year-old Hispanic male presented to the emergency department with the chief concern of shortness of breath and abdominal pain. Incidentally, an X-ray demonstrated multiple round hyperdense foreign bodies in the ileum and cecum. Physical exam was positive for right-sided and periumbilical abdominal pain without any peritoneal signs. Upon colonoscopy, 14 hearing aid batteries of size 312 were discovered without evidence of perforation or obstruction. Ingestion of batteries in adults is a rare phenomenon. When an adult presents with ingestion of dangerous foreign bodies such as batteries, mental health is critical to consider in the history and treatment plan.

## Introduction

Foreign body ingestion is a relatively common occurrence in children between the ages of six months and six years, whereas it is rarely seen in adults. When it does occur, the most ingested objects are chicken and fish bones [1-3]. In most cases, the foreign body passes through the digestive tract without intervention, but 20% of the time, imaging through esophagogastroduodenoscopy or colonoscopy and/or surgery is required for removal [3]. In some cases, ingested foreign bodies can obstruct, becoming lodged in the upper, middle, and lower esophagus. When this occurs, urgent intervention is vital owing to the potential for mucosal irritation and destruction, especially with corrosive batteries [4,5]. While those kinds of ingestions are mainly accidental, deliberate foreign body ingestion (DFBI) may occur in patients with mental health issues, such as depression and borderline personality disorder. More than half of these patients present with frequent recurrence of DFBI [1,4].

## Case presentation

**Figure 1:**
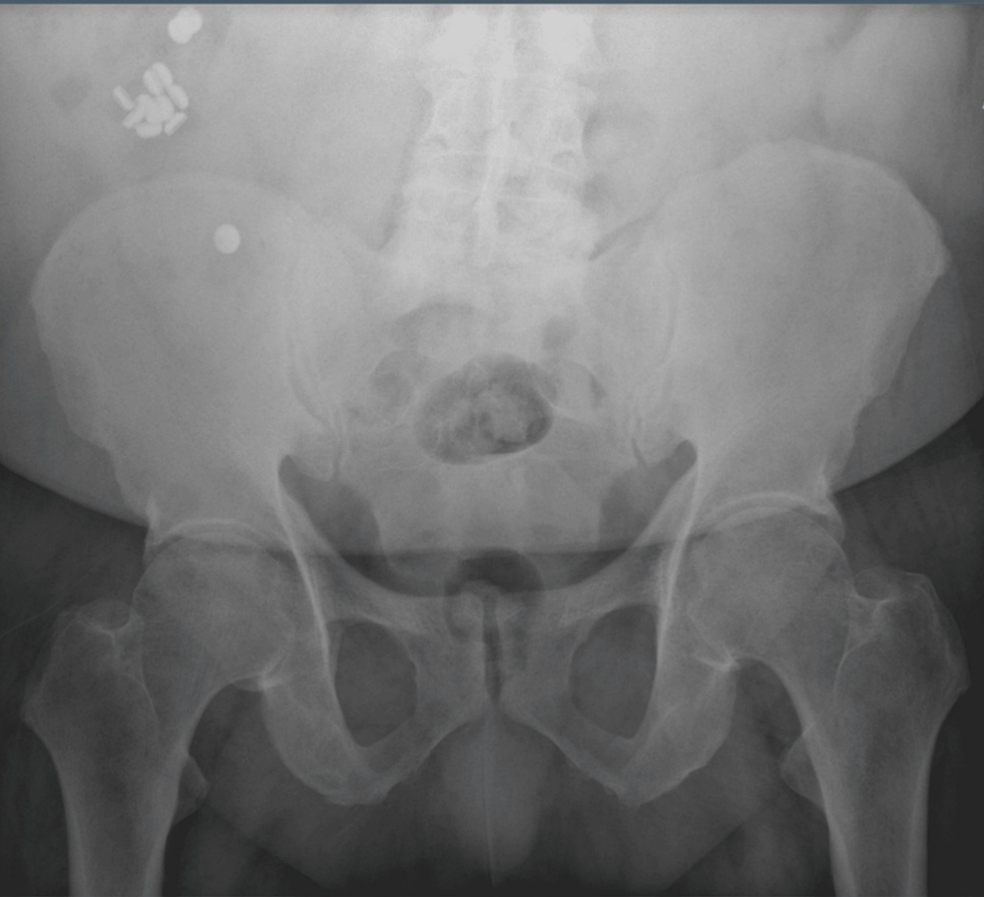
Multiple round hyperdense objects in the intestines on the radiograph

**Figure 2:**
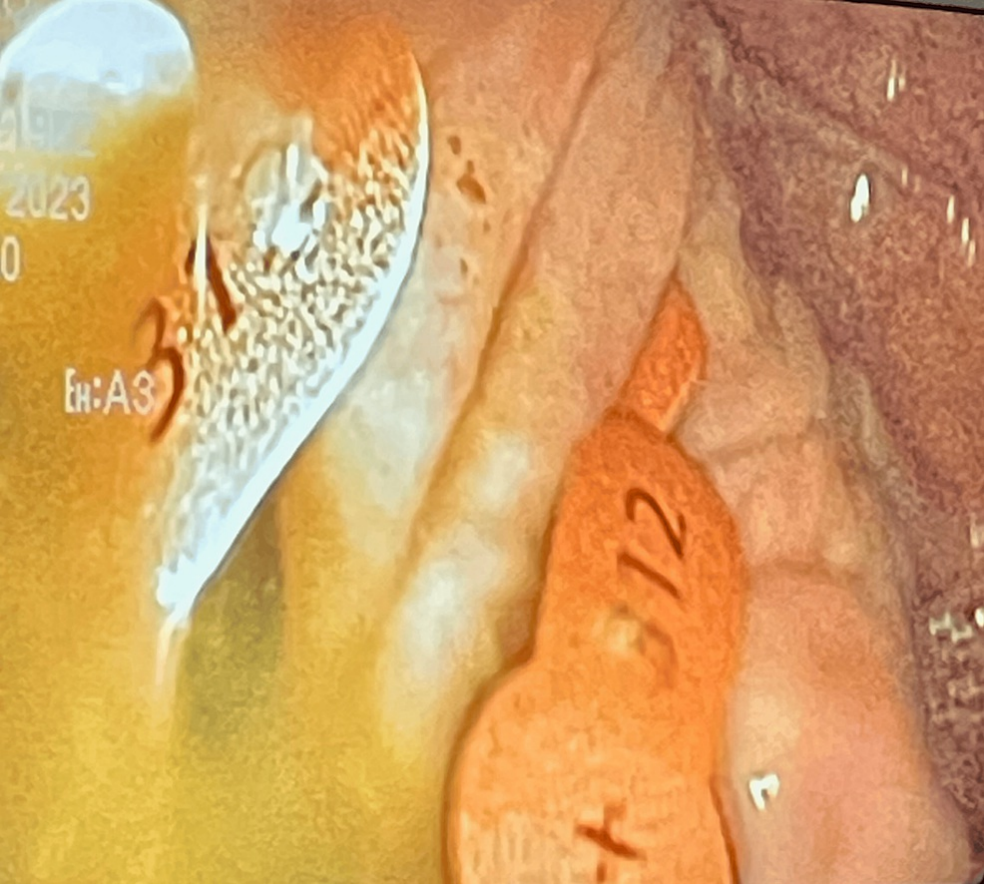
Multiple 312 batteries seen in the ileum on colonoscopy

**Figure 3:**
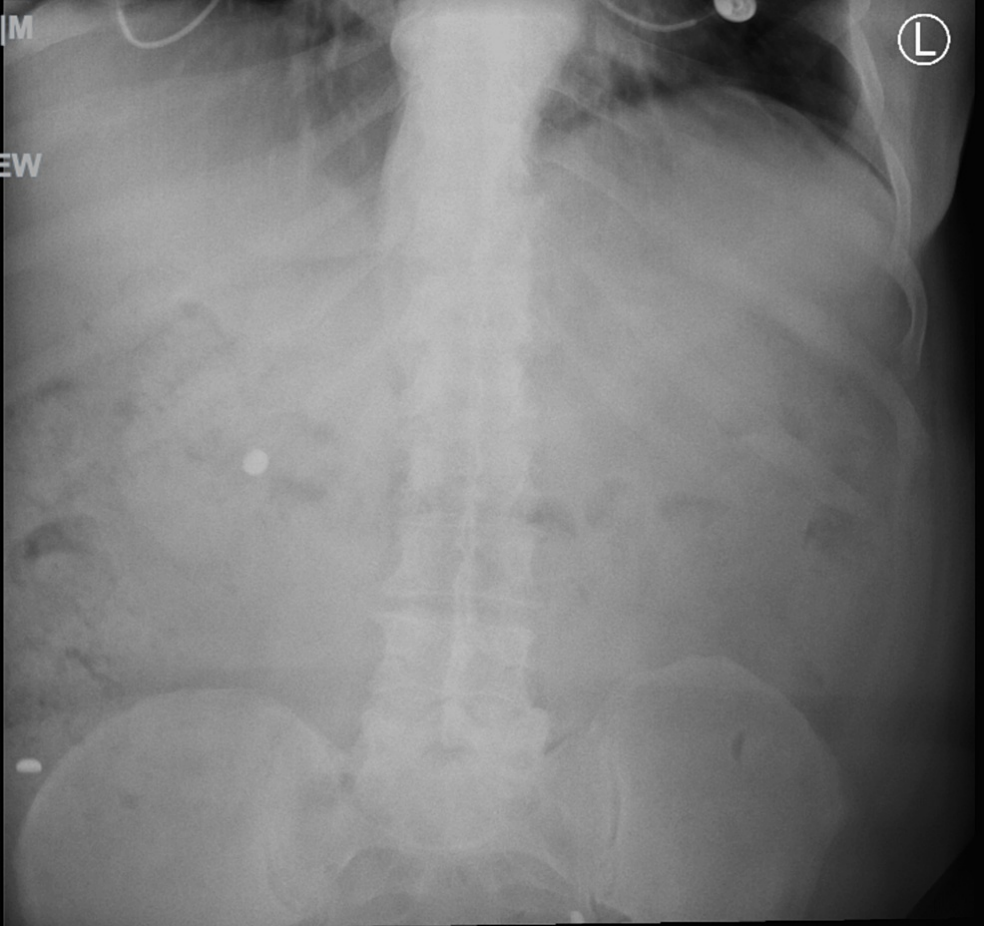
More hyperdense round objects seen on the radiograph one day after the colonoscopy

**Figure 4:**
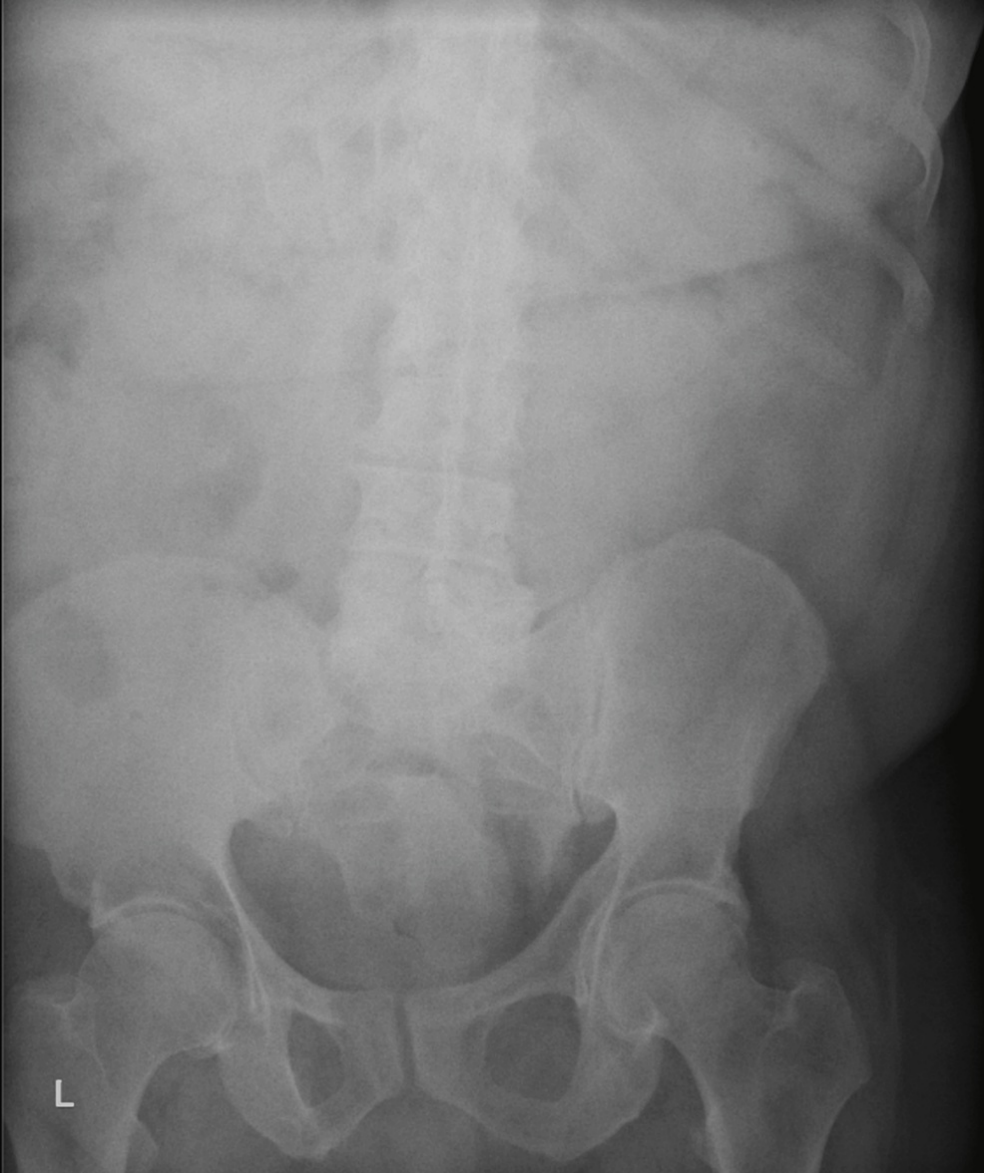
Radiograph after the passage of all batteries

A 61-year-old male with a past medical history of asthma, congestive heart failure (CHF), chronic untreated depression, chronic obstructive pulmonary disease (COPD), coronary artery disease, gastroesophageal reflux disease (GERD), hyperlipidemia, hypertension, and tracheobronchial malacia presented to the emergency department, complaining of shortness of breath and abdominal pain. He arrived after being released from a different hospital the previous day for flu-like symptoms. Of note, he had been admitted previously to the same hospital a month prior because of shortness of breath, which cardiac workup and chest imaging showed no abnormalities. After endorsing intense 10/10 abdominal pain on the current encounter, he had a CT scan of the abdomen/pelvis that revealed two round metal densities in his distal ileum without evidence of bowel obstruction. He refused a colonoscopy at the time because of cultural reasons and left against medical advice. On current admission, his physical exam revealed a painful abdominal exam and bilateral lower lobe wheezing. He tested positive for Influenza A and received oseltamivir and Tylenol for his symptoms. Although the cause of his shortness of breath was likely from his underlying influenza diagnosis, his continuing abdominal pain prompted more imaging and further investigation. An abdomen X-ray was ordered, showing more than two round metal densities in his distal ileum and cecum (Figure 1). The patient did not give any insight into the ingestion and denied any knowledge of swallowing anything metallic, stating that he frequents fast-food restaurants. Moreover, he explained that he was a political prisoner in Cuba for six years and underwent major stressors but did not go into more depth on that topic any further. Based on these findings, gastrointestinal was consulted, and he consented to a colonoscopy, during which 14 312 hearing aid batteries were discovered in the ileum and cecum (Figure 2). All 14 batteries were removed via colonoscopy, and there was no mucosal damage or bowel injury evident. On the next day, follow-up radiography of the kidney, ureters, and bladder showed more metallic foreign bodies that were not seen on colonoscopy; however, they resolved spontaneously in the following days (Figures 3-4). The patient's flu symptoms, mood, and abdominal pain resolved, and the patient was discharged without complications a few days later. On discharge, he was prescribed 5 mg po daily escitalopram, and it was recommended that he pursue outpatient psychotherapy.

## Discussion

Given the rarity of foreign body ingestion in adults, this case illustrates the importance of using past medical history to guide diagnosis and treatment in unconventional patient populations such as this one. Although children comprise 80% of patients presenting with foreign body ingestion, medical professionals should still consider it as a possibility in adults when the presentation and past medical history make it more likely. Some complications of battery ingestion include mucosal burns, perforation, formation of tracheoesophageal fistula, major hemorrhage, and even death [5]. In children, the complication rate is 0.8%, and the mortality rate is 0.15% [6]. Furthermore, various psychiatric issues such as depression, pica, borderline personality disorder, and so on may contribute to this unusual presentation in adults such as in this case where there was a history of depression, an extensive problem list, and multiple hospitalizations [1,4]. This case simultaneously highlights the importance of considering mental health when gathering patient history, making differential diagnoses, and forming a plan. Interestingly, the patient denied knowing how he ingested these batteries and went on to acquire even more during his hospital stay. Equipping inpatient medical professionals to better recognize signs of mental distress among routine clinical presentations, such as shortness of breath and abdominal pain, would improve the time to diagnosis, time to intervention, and improve patient outcomes [1].

## Conclusions

This case is salient in highlighting the importance of assessing the mental health status in the event of foreign body ingestion in adults, especially those with a past medical history of depression. It is a reminder that clinical presentations more common in pediatric patients should not always be ruled out in adults.
